# The role of ORMDL3/ATF6 in compensated beta cell proliferation during early diabetes

**DOI:** 10.18632/aging.101949

**Published:** 2019-05-06

**Authors:** Weixia Yang, Feifei Sheng, Baolan Sun, Shane Fischbach, Xiangwei Xiao

**Affiliations:** 1 Department of Pediatrics, Affiliated Hospital of Nantong University, Nantong 226001, China; 2 Department of Surgery, Children’s Hospital of Pittsburgh, University of Pittsburgh School of Medicine, Pittsburgh, PA 15224, USA

**Keywords:** ER stress, unfolded protein response (UPR), beta cell proliferation, ORMDL3, ATF6

## Abstract

Endoplasmic reticulum (ER) stress in beta cells induces a signaling network called the unfolded protein response (UPR), which plays a dual role in diabetes. A key regulator of ER-stress and UPR, the orosomucoid 1-like protein 3 (ORMDL3), has been shown to regulate airway remodeling through a major UPR protein, activating transcription factor 6 (ATF6), but the contribution of this regulatory axis to compensatory pancreatic beta cell proliferation in diabetes has not been studied. Here, we detected significantly lower levels of ORMDL3 mRNA in leukocytes of peripheral blood specimens from type 1 diabetes (T1D) children, compared to normal children. Moreover, these ORMDL3 levels in T1D children exhibited further decreases upon follow-up. ORMDL3 levels in islets from NOD mice, a mouse model for T1D in humans, showed a mild increase before diabetes onset, but a gradual decrease subsequently. In high glucose culture, beta cell proliferation, but not apoptosis, was increased by overexpression of ORMDL3 levels, likely mediated by its downstream factor ATF6. Mechanistically, ORMDL3 transcriptionally activated ATF6, which was confirmed in a promoter reporter assay. Together, our data suggest that ORMDL3 may increase beta cell proliferation through ATF6 as an early compensatory change in response to diabetes.

## INTRODUCTION

Diabetes is a prevalent metabolic disease characterized by high blood sugar caused by either inadequate insulin production by beta cells in the pancreas, or by a defective response to insulin in the liver, muscle and adipose tissue [1]. There are two types of diabetes, type 1 diabetes (T1D) and type 2 diabetes (T2D), which both result in a loss of functional beta cell mass despite different etiologies and pathological processes [2].

Endoplasmic reticulum (ER) stress is often generated by the expression of mutant proteins that impair normal protein folding in ER, a process which also involves a signaling network called the unfolded protein response (UPR) [3]. The UPR upregulates ER chaperone, foldase and protein secretion-associated genes, leading to a healthy augmentation of the biosynthetic capacity of the secretory pathway [4]. ER stress plays a pivotal role in the pathogenesis of diabetes, contributing to both the loss of functional beta cells and the development of insulin resistance [5]. UPR, however, appears to play a dual role in beta cells, being beneficial to cells under physiological conditions or mild stress, but detrimental to cells during pathological stages or chronic stress by triggering beta-cell dysfunction and apoptosis [6].

The orosomucoid 1-like protein 3 (ORMDL3) is one of three members of the ORDML gene family (ORMDL-1, ORMDL-2, and ORMDL3), and is an ER-resident transmembrane protein that is 96% homologous between humans and mice. ORMDL3 is expressed in the adult lung, liver, kidney and pancreas [7, 8]. An association between ORMDL3 and asthma has been reported in a genome-wide association study [7], and later studies have shown that ORMDL3 may regulate airway remodeling through activating transcription factor 6 (ATF6), a critical transmembrane sensor for ER stress, and its downstream gene SERCA2b [9, 10], or through transforming growth factor β (TGF-β) [10] or metalloproteases [9]. Moreover, the regulatory axis ORMDL3/ATF6 has been shown to regulate splenic B cell survival through Beclin-1 [11].

Very recently, a role for UPR in diabetes and the regulation of pancreatic beta cell proliferation has been reported. First, tauroursodeoxycholic acid (TUDCA), an ER stress/UPR controller, protected mice from developing T1D through restoration of the UPR in beta cells, attributable to the regulatory function of UPR in autophagy and apoptotic pathways in beta cells [12]. In another study, UPR has been found to be a mediator of insulin demand on beta cell proliferation [13]. The latter study is extremely important, since we and others have shown that increases in beta cell number in adults, especially in situations like pregnancy and transient hyperglycemia, preliminarily result from beta cell self-replication [14–18]. Given the fact that it is very difficult to guide adult beta cells into an active cell cycle [19–25], studying the mechanisms underlying UPR regulation of beta cell proliferation in early diabetes is very important and may provide useful information for the early treatment of diabetes.

Here, we detected significantly lower levels of ORMDL3 mRNA in leukocytes of peripheral blood specimens from T1D children, compared to normal children. Moreover, these ORMDL3 levels in T1D children exhibited further decreases upon follow-up. ORMDL3 levels in islets from NOD mice showed a mild increase before diabetes onset, but a gradual decrease subsequently. In high glucose culture, overexpression of ORMDL3 in a mouse beta cell line (Min6) significantly increased ATF6 and beta cell proliferation, without altering apoptosis. Depletion of ORMDL3 in Min6 significantly decreased ATF6 and beta cell proliferation, without altering apoptosis. On the other hand, overexpression of ATF6 increased beta cell proliferation, without altering ORMDL3 levels or apoptosis, while depletion of ATF6 decreased beta cell proliferation, without altering ORMDL3 levels or apoptosis. Suppression of ATF6 abolished the effects of overexpression of ORMDL3 on cell proliferation. Mechanistically, ORMDL3 activated ATF6 transcriptionally in a promoter reporter assay. Together, our data suggest that ORMDL3 may increase beta cell proliferation through ATF6 as a beta-cell compensation against diabetes.

## RESULTS

### ORMDL3 mRNA decreases in leukocytes of peripheral blood specimens from T1D children

**Figure 1:**
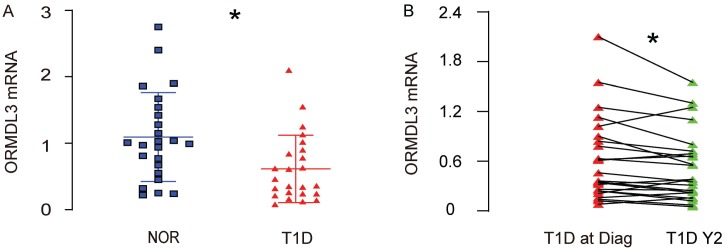
**ORMDL3 mRNA decreases in leukocytes of peripheral blood specimens from T1D children.** (**A**) RT-qPCR for ORMDL3 in leukocytes of peripheral blood specimens from 24 T1D children and 24 normal children (NOR). (**B**) RT-qPCR for ORMDL3 in leukocytes of peripheral blood specimens from 24 T1D children, at time of diagnosis (T1D at Diag) and 2 years after diagnosis (T1D Y2). *p<0.05. N=24.

A key regulator of ER-stress and UPR, the orosomucoid 1-like protein 3 (ORMDL3), has been shown to regulate airway remodeling through a major UPR protein, activating transcription factor 6 (ATF6), but the contribution of this regulatory axis on compensatory pancreatic beta cell proliferation in diabetes has not been studied. Here, we addressed this question. We isolated leukocytes of peripheral blood specimens from 24 T1D children and 24 normal children (Table 1). We detected significantly lower levels of ORMDL3 mRNA in leukocytes of peripheral blood specimens from T1D children, compared to normal children (Figure 1A). Moreover, all the T1D children received a second measurement for ORMDL3 mRNA in leukocytes of peripheral blood specimens 2 years after the first measurement. The ORMDL3 levels in these 24 T1D children exhibited significant decreases (Figure 1B). These data suggest that ORMDL3 mRNA may gradually decrease in leukocytes of peripheral blood specimens from T1D children.

**Table 1:** Clinical characteristics (total)

	**T1D Patients (24)**	**Normal (24)**	**P value**
Age (yrs)	16.2±3.5	15.3±3.8	0.55
Age of T1D onset (yrs)	13.7±2.3	n/a	
Duration of disease (yrs)	2.6±1.9	n/a	
Gender (male/female)	15 (62.5%) /9 (37.5%)	13 (54.2%) /11 (45.8%)	0.56
Fasting blood glucose (mg/dl)	193.7±62.2	87.5±13.6	<0.0001
HbA1C level	10.3±4.5%	4.8±0.7%	<0.0001

### ORMDL3 levels slightly increase in NOD mouse islets before diabetes onset, but continue decreasing after diabetes onset

**Figure 2:**
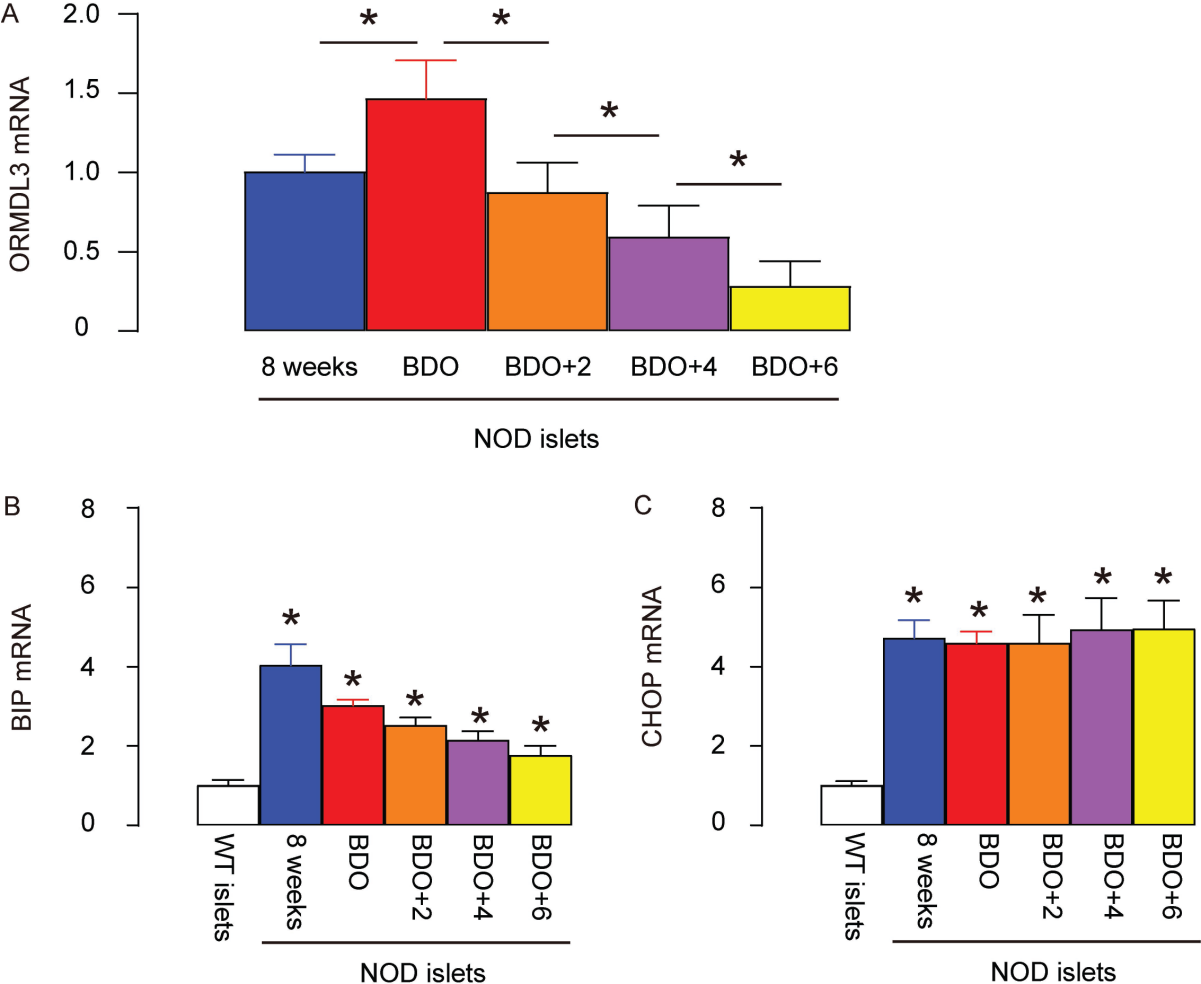
**ORMDL3 levels slightly increase in NOD mouse islets before diabetes onset, but continue decreasing after diabetes onset**. (**A–C**) RT-qPCR for ORMDL3 (**A**), BIP (**B**) and CHOP (**C**) in islets from female NOD mice, at 8 weeks of age (8 weeks), the first time when fasting blood glucose reaches 150mg/dl (before diabetes onset, BDO), and 2 or 4 or 6 weeks after BDO. WT: wildtype female C57BL/6 mice at 8 weeks of age. *p<0.05. NS, no significance. N=5.

Next, we aimed to examine the changes in ORMDL3 in beta cells at diabetes onset. Thus, we selected NOD mice to study, since NOD mice are the most widely used model for T1D. Since female NOD mice have a much higher chance of developing diabetes, we used female mice in the current study. Two important time courses were selected. The first stage was at 8 weeks of age, when all the mice exhibit normoglycemia. At this stage, although insulitis should be mild, beta cells are alive and function properly. The second stage was the first time when fasting blood glucose reached 150mg/dl at around 14 weeks of age. Based on our experience, whenever the fasting blood glucose level of the NOD mouse reaches 150mg/dl, it may start to increase very quickly and the condition of the mouse drops precipitously. This time course was named “beginning of diabetes onset” (BDO). We compared the ORMDL3 mRNA levels in islets from 8-week-old NOD mice, at BDO, 2 weeks after BDO (BDO+2), 4 weeks after BDO (BDO+4) and 6 weeks after BDO (BDO+6). The later time courses were not included due to the high mortality of the mice. We found that ORMDL3 levels slightly increased in NOD mouse islets at BDO, compared to at 8-weeks-old, but continued decreasing after BDO with time (Figure 2A). Binding of immunoglobulin protein (BIP) and CCAAT/enhancer-binding protein homologous protein (CHOP) are two of the best studied genes transcriptionally induced by ER stress. We examined BIP and CHOP levels in these samples, and found that they were both upregulated in the islets from NOD mice, as early as before diabetes onset, compared to control wildtype C57BL/6 mice (Figure 2B, 2C). Together, these data confirm the presence of ER stress in NOD mice, and are consistent with previous reports showing a dual role of UPR in diabetes.

### ATF6 increases beta cell proliferation cultured in HG

**Figure 3:**
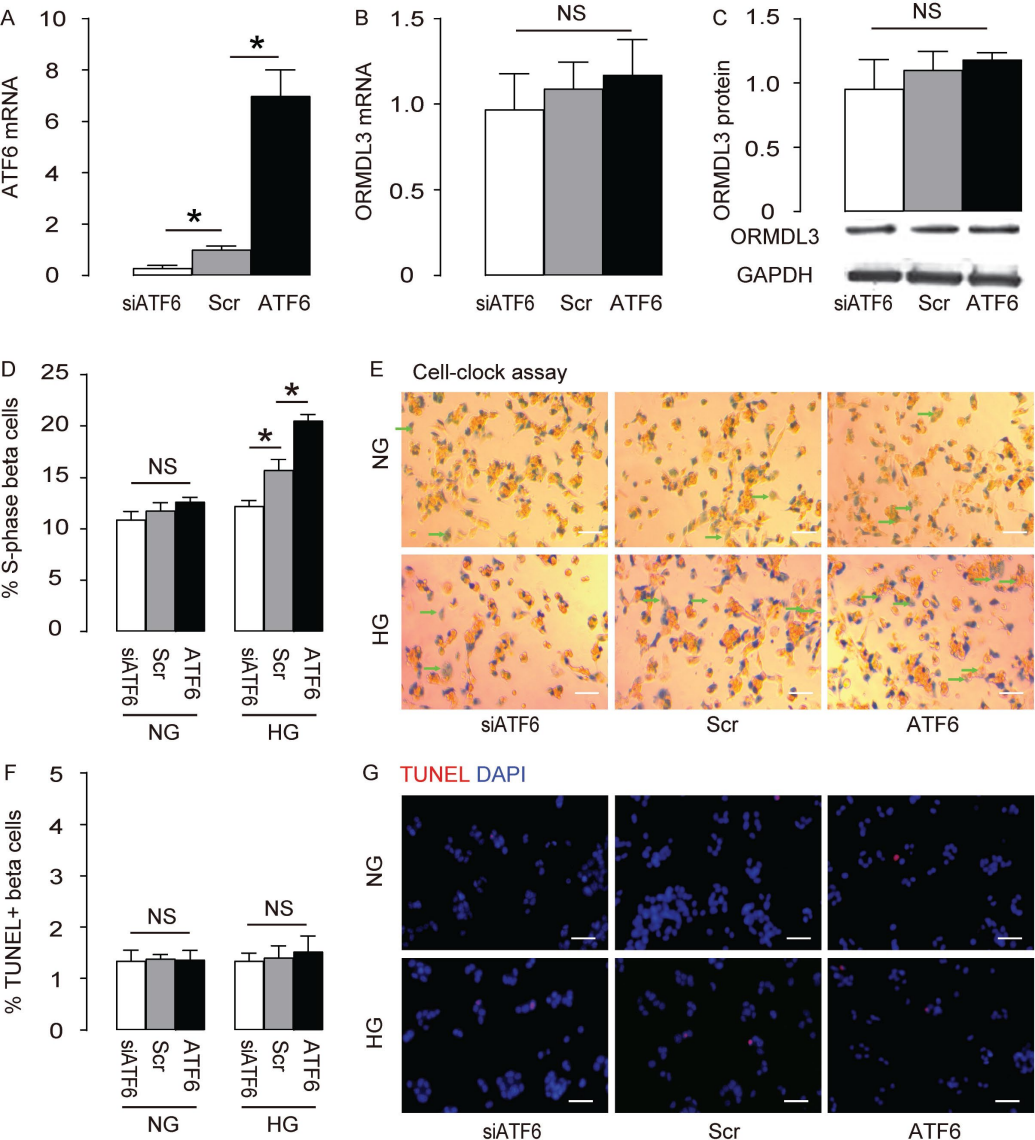
**ATF6 increases beta cell proliferation cultured in HG. Min6 cells were kept in high glucose (20mmol/l) culture, and transfected with ATF6, or scrambled (Scr), or siRNA for ATF6 (siATF6).** (**A–B**) RT-qPCR for ATF6 (**A**) and ORMDL3 (**B**). (**C**) Western blot for ORMDL3. (**D–E**) Cell-clock cell cycle assay, shown by quantification of S-phase cells (**D**), and by representative images (**E**). (**F–G**) TUNEL assay, shown by quantification (**F**), and by representative images (**G**). DAPI: nuclear staining. NG: normal glucose culture. HG: high glucose culture. Arrows pointed to S-phase cells. *p<0.05. NS, no significance. N=5. Scale bars are 20μm.

We are interested in assessing the role of ORMDL3 before diabetes onset, since it may provide some novel mechanisms to resist the development of diabetes. ATF6 is the most important target of ORMDL3, so we examined the effects of altering ATF6 levels on ORMDL3 and cell proliferation in beta cells. We either overexpressed ATF6 by transfection of ATF6-coding sequence, or depleted ATF6 by siATF6 in Min6 cells, and used scrambled (Scr) transfection as a control. First, the alteration of ATF6 mRNA by transfection of ATF6, siATF6 or Scr was confirmed (Figure 3A). However, alteration of ATF6 did not alter ORMDL3 mRNA levels (Figure 3B) and protein levels (Figure 3C), suggesting that ATF6 does not control ORMDL3. We cultured the mouse beta cell line Min6 in either normal glucose (NG) or high glucose (HG), to determine if high glucose may exert a stress on Min6 cells. We found that overexpression of ATF6 significantly increased cell proliferation in HG but not in NG, shown by quantification (Figure 3D), and by representative images (Figure 3E), while depletion of ATF6 significantly decreased cell proliferation in HG but not in NG, shown by quantification (Figure 3D), and by representative images (Figure 3E). Alteration of ATF6 levels did not affect levels of cell apoptosis in either HG or NG (Figure 3F–3G). Together, these data suggest that ATF6 increases beta cell proliferation in HG, but does not change apoptosis.

### ORMDL3 increases beta cell proliferation cultured in HG through ATF6

**Figure 4:**
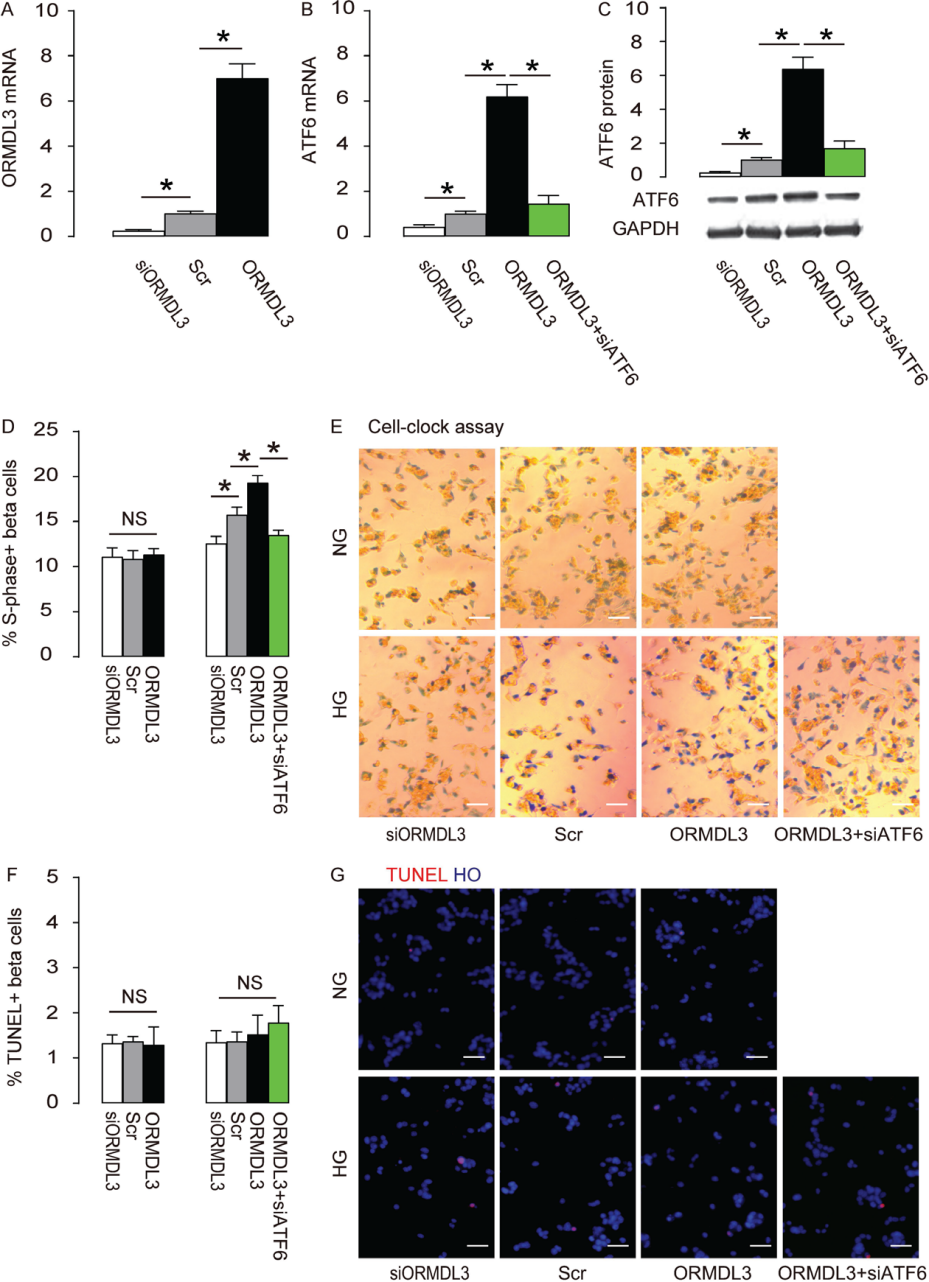
**ORMDL3 increases beta cell proliferation cultured in HG through ATF6. Min6 cells were kept in high glucose (20mmol/l) culture, and transfected with ORMDL3, or scrambled (Scr), or siRNA for ORMDL3 (siORMDL3) or ORMDL3 plus siATF6.** (**A–B**) RT-qPCR for ORMDL3 (**A**) and ATF6 (**B**). (**C**) Western blot for ATF6. (**D–E**) Cell-clock cell cycle assay, shown by quantification of S-phase cells (**D**), and by representative images (**E**). (**F–G**) TUNEL assay, shown by quantification (**F**), and by representative images (**G**). DAPI: nuclear staining. NG: normal glucose culture. HG: high glucose culture. *p<0.05. NS, no significance. N=5. Scale bars are 20μm.

Next, we examined the effects of altering ORMDL3 levels on ATF6 and cell proliferation in beta cells. We either overexpressed ORMDL3 by transfection of ORMDL3-coding sequence, or depleted ORMDL3 by siORMDL3 in Min6 cells, and used Scr transfection as a control. First, the alteration of ORMDL3 mRNA by transfection of ORMDL3, siORMDL3 or Scr was confirmed (Figure 4A). We found that overexpression of ORMDL3 significantly increased ATF6 mRNA (Figure 4B) and protein (Figure 4C), while depletion of ORMDL3 significantly decreased ATF6 mRNA (Figure 4B) and protein (Figure 4C), suggesting that ORMDL3 controls ATF6. We cultured the mouse beta cell line Min6 in either NG or HG. We found that overexpression of ORMDL3 significantly increased cell proliferation in HG but not in NG, shown by quantification (Figure 4D), and by representative images (Figure 4E), while depletion of ORMDL3 significantly decreased cell proliferation in HG but not in NG, shown by quantification (Figure 4D), and by representative images (Figure 4E). Alteration of ORMDL3 levels did not affect levels of cell apoptosis in either HG or NG (Figure 4F–4G). In order to examine whether the effects of ORMDL3 on beta cell proliferation are mediated by ATF6, we depleted ATF6 in ORMDL3-overexpressing beta cells, and confirmed it by RT-qPCR (Figure 4B) and by Western blot (Figure 4C). We found that ATF6 depletion abolished the effects of ORMDL3 on beta cell proliferation in HG (Figure 4D–4E), without affecting apoptosis (Figure 4F–4G). Thus, ORMDL3 increases beta cell proliferation cultured in HG through ATF6.

### ORMDL3 transcriptionally activates ATF6 in beta cells

**Figure 5:**
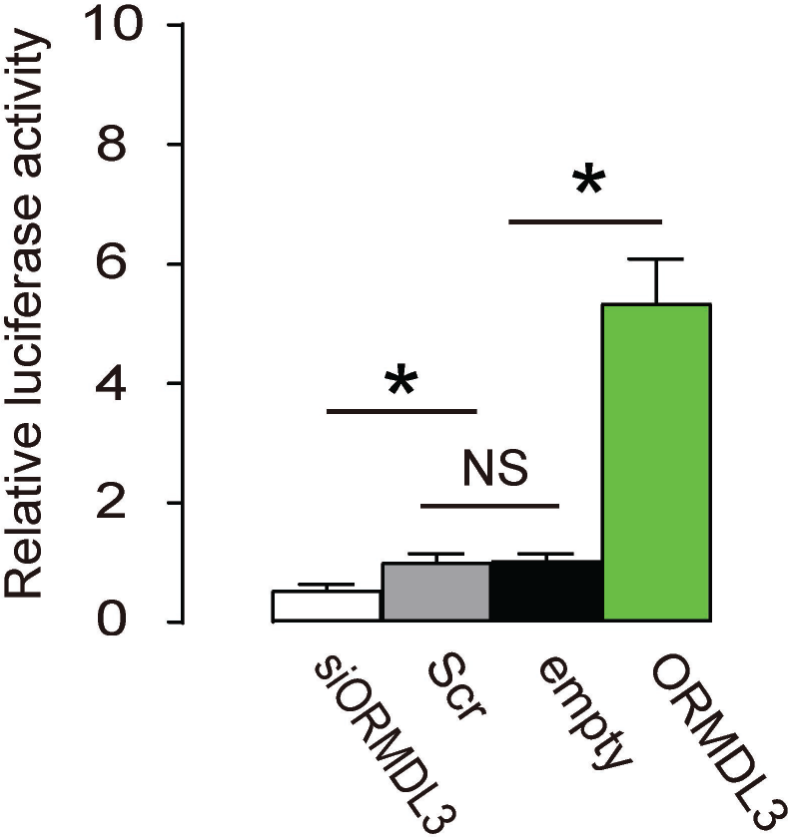
**ORMDL3 transcriptionally activates ATF6 in beta cells.** An ATF6 Reporter Assay was applied to Min6 cells, using co-transfection of the reporter and ORMDL3, or siORMDL3 or Scr or empty. Luciferase activity was determined. *p<0.05. NS, no significance. N=5.

Finally, an ATF6 Reporter Assay was applied to Min6 cells, using co-transfection of the reporter and ORMDL3, or siORMDL3 or Scr or empty. We found no difference in luciferase activity between Scr and empty. However, co-transfection with ORMDL3 significantly increased the luciferase activity by 5.3 folds, while co-transfection with siORMDL3 significantly decreased luciferase activity to 45% (Figure 5). These data suggest that ORMDL3 transcriptionally activates ATF6 in beta cells.

## DISCUSSION

The ER plays a central role in the synthesis, folding and trafficking of cellular proteins to their respective targets. Hyperglycemia in diabetes provokes production of mitochondrial reactive oxygen species (ROS) and protein glycation, leading to an exaggerated ER load of misfolded/unfolded and nascent proteins associated with insulin production and release, which impairs ER homeostasis and causes ER stress [26]. ER stress induces activation of UPR, which mediates oligomerization and cross-autophosphorylation to rebalance ER homeostasis and restore the proper function of the ER. However, sustained ER stress and activation of UPR may activate autophagy and apoptotic pathways to cause beta cell death [27]. Some previous studies have demonstrated the contribution of IRE1/XBP1 [28] and PERK-eIF2α pathways [29] in beta-cell apoptosis and insulitis, leading to the development of T1D.

In a very recent report, the role of UPR has been associated with pro-diabetic beta cell proliferation [13]. Since we and others have shown that increases in beta cell number in adults, especially in some situations like pregnancy, transient hyperglycemia, preliminarily result from beta cell self-replication [14–18], it is very important to know that UPR may increase beta cell proliferation, possibly as a reactive response to high insulin demand and workload on ER in this short time window. Proper utilization of this time window to amplify or sustain the beneficial effects of UPR may greatly help to prevent diabetes onset. It is noteworthy that our hypothesis was based on several previous studies. First, insulin demand triggers UPR to increase beta cell proliferation, in which ATF6 plays a critical role [13]. Second, ORMDL3 regulates airway remodeling through ATF6 and its downstream gene SERCA2b [9, 10]. Finally, one study from our own group has shown that ubiquitin ligase Cbl-b inhibits human ORMDL3 expression through STAT6 [36]. To summarize these previous studies, it is an obvious question whether the ORMDL3/ATF6 regulatory axis plays a role in insulin demand-induced beta cell proliferation, which was addressed here, where we focused on the initial activation of UPR and associated proteins ORMDL3 and ATF6 in a pro-diabetic environment, using 3 days’ culture in high glucose. Very modest apoptosis was detected in the experiments.

Both ORMDL3 and ATF6 are ER-harboured membrance proteins, but our promoter assay suggests that the regulation of ORMDL3 on ATF6 in beta cells is mediated at least partially through direct promoter binding and the subsequent activation of ATF6 gene expression. The regulation of ORMDL3 on ATF6 may also include post-transcriptional control, given the close positioning of the proteins. This question may be addressed in future studies. The absence of the regulation of ATF6 on ORMDL3 suggests a lack of positive or negative feedback on the regulation of ATF6 on ORMDL3, which may be responsible for the relatively weak and unstable regulation of ORMDL3-mediated UPR on beta cell proliferation, since it is overwhelmed by the activation of autophagy and apoptosis pathways in a longer time course [27].

The lesson from the current study is that there is a relatively short timeframe in which activated UPR in pro-diabetic mice indeed has a beneficial effect on the body, possibly through augmentation of beta cell proliferation to compensate for the sudden increase in insulin production. The slowness of beta cell proliferation limits the net effects of UPR, the failure of which leads to apoptotic beta cell death. Effective amplification of this early UPR and slowdown of ER stress may be important for beta cell protection against ER-stress-induced damage.

## MATERIALS AND METHODS

### Protocol ethics issues and mouse treatment

All animal experiments were approved by the Animal Research and Care Committee at Nantong University. Female non-obese diabetic (NOD) mice of 6 weeks of age and female C56BL/6 mice of 6 weeks of age were both purchased from the SLAC Laboratory Animal Co. Ltd (Shanghai, China). Fasting blood glucose levels were measured at around 10am by tail tip puncture with a glucometer (Accu-Chek, Roche, Nutley, NJ, USA) after 16 hours’ overnight fasting, as has been previously described [30]. For the use of clinical information and specimens for research purposes, prior patients’ consent and approval from the Institutional Research Ethics Committee of Nantong University were obtained. All cases were clinically diagnosed at the Affiliated Hospital of Nantong University from 2009 to 2016 (Table 1). Individuals who had other diseases were excluded from the current study.

### Culture and transfection of beta cell line Min6

Min6 is a mouse insulinoma cell line, which was purchased from American Type Culture Collection (ATCC, Rockville, MD, USA), and grown in normal (5mmol/l) or high (20mmol/l)-glucose Dulbecco’s Modified Eagle’s Medium (DMEM, Invitrogen, Carlsbad, CA, USA) supplemented with 10% fetal bovine serum (FBS; Sigma-Aldrich, St Louis, MO, USA) in a humidified chamber with 5% CO_2_ at 37 °C. ORMDL3 and ATF6 coding constructs were cloned from mouse lung cDNA. Small interfering RNA (siRNA) for ORMDL3 and ATF6 were obtained from OriGene Technologies, Inc. (Catalog number: SR403232 and SR418766; Rockville, MD, USA). The plasmids for generating these constructs used a pcDNA3.1-CMV-GFP plasmid as a backbone (Addgene, Mountain View, CA, USA; Catalog number: 11153), and restriction enzymes Kpnl and Notl as cloning sites. Analysis was done 72 hours after transfection of cells with a Lipofectamine-3000 assay (Invitrogen, Shanghai, China).

### Assessment of cell proliferation and apoptosis

Cell proliferation was measured with a Cell-clock cell cycle assay, which uses a redox dye that is imported by live cells and exhibits particular color changes associated with cells in the G1 (small, pale yellow and circular in shape), S (large, light green and fibroblast-like in shape), G2 (dark green and round in shape) and M (intense blue and round in shape) phases of the cell cycle to assess cell proliferation (Biocolor LTD, Carrickfergus, UK). In the current study, the ratio of S-phase cells over total cells was used to evaluate cell proliferation.

Terminal deoxynucleotidyltransferase-mediated dUTP-biotin nick end labeling (TUNEL) staining was performed with an ApopTag® Peroxidase In Situ Apoptosis Detection Kit (Millipore, Billerica, MA, USA), according to the manufacturer’s instructions, as previously described [31].

### Pancreatic digestion and islet isolation

Mouse pancreas digestion and islet isolation were performed as described before [32–35]. Briefly, the pancreas was infused with 0.125 mg/ml Liberase (Sigma-Aldrich, St. Louis, MO, USA) for 40 minutes to obtain a single cell population. Islets were handpicked at least 5 times to avoid contamination of non-islets.

### RT-qPCR

RT-qPCR was performed using the QuantiTect SYBR Green PCR Kit (Qiagen, Shanghai, China), with the primer pairs for ORMDL3: Fwd: CCCTCACCAACCTTATCCAC and Rev: GGACCCCGTAGTCCATCTG; for ATF6: Fwd: GGACGAGGTGGTGTCAGAG and Rev: GACAGCTCTTCGCTTTGGAC; for BIP: Fwd: CCTGCGTCGGTGTGTTCAAG and Rev: AAGGGTCATTCCAAGTGCG; for CHOP: Fwd: ACGGAAACAGAGTGGTCAGTGC and Rev CAGGAGGTGATGCCCACTGTTC; and for GAPDH: Fwd: AACTTTGGCATTGTGGAAGG and Rev: ACACATTGGGGGTAGGAACA. A 2^-△△Ct^ method was used for quanti-fication of gene expression levels. Relative expression levels of genes were obtained through sequential normalization of the values against GAPDH and experimental controls.

### ATF6 reporter assay kit

An ATF6 Reporter Assay Kit (Qiagen; Catalog number: CCS-9031L) was used to measure the transcriptional activity of ATF6. The ATF6 reporter is a mixture of an ATF6-responsive luciferase construct and a constitutively expressing Renilla luciferase construct (40:1). The ATF6-responsive luciferase construct encodes the firefly luciferase reporter gene under the control of a minimal CMV promoter and tandem repeats of the ATF6 transcriptional response element. The ATF6 reporter construct monitors both increases and decreases in the transcriptional activity of ATF6, and hence the activity of hedgehog signaling.

### Western blotting

Western blotting was performed as previously described [36]. Primary antibodies for Western blot are rabbit anti-GAPDH (1:1000; Cell signaling, San Jose, CA, USA; Catalog number: #2118), rabbit anti-ORMDL3 (1:500; Abcam; Catalog number: ab107639), and rabbit anti-ATF6 (1: 1000; Cell signaling; Catalog number: #65880). The secondary antibody is HRP-conjugated anti-rabbit antibody (Jackson ImmunoResearch Labs, West Grove, PA, USA).

### Data analysis

GraphPad Prism 7 (GraphPad, Chicago, IL, USA) software was used to analyze data in the current study. Statistical analysis on clinical specimens was performed using the Wilcoxon Test, while data from in vitro studies were statistically analyzed using a stringent multiple comparison post hoc test to compare data in multiple groups, or one-way ANOVA with a Bonferroni correction, followed by Fisher’s Exact Test for comparison between two groups. All values are depicted as mean ± standard deviation and are considered significant if p < 0.05.
